# Layer 6 is a hub for cholinergic modulation in the mouse auditory cortex

**DOI:** 10.1093/cercor/bhaf338

**Published:** 2026-01-22

**Authors:** Lucas G Vattino, Kameron K Clayton, Troy A Hackett, Daniel B Polley, Anne E Takesian

**Affiliations:** Eaton-Peabody Laboratories, Massachusetts Eye and Ear, 243 Charles St, Boston, MA 02114, United States; Department of Otolaryngology - Head and Neck Surgery, Harvard Medical School, 25 Shattuck St, Boston, MA 02115, United States; Eaton-Peabody Laboratories, Massachusetts Eye and Ear, 243 Charles St, Boston, MA 02114, United States; Department of Otolaryngology - Head and Neck Surgery, Harvard Medical School, 25 Shattuck St, Boston, MA 02115, United States; Vanderbilt School of Medicine, Department of Hearing and Speech Sciences, Vanderbilt University Medical Center, 1211 Medical Center Dr, Nashville, TN 37232, United States; Eaton-Peabody Laboratories, Massachusetts Eye and Ear, 243 Charles St, Boston, MA 02114, United States; Department of Otolaryngology - Head and Neck Surgery, Harvard Medical School, 25 Shattuck St, Boston, MA 02115, United States; Eaton-Peabody Laboratories, Massachusetts Eye and Ear, 243 Charles St, Boston, MA 02114, United States; Department of Otolaryngology - Head and Neck Surgery, Harvard Medical School, 25 Shattuck St, Boston, MA 02115, United States

**Keywords:** acetylcholine, basal forebrain, layer 6 corticothalamic neurons, neuromodulation, nicotinic and muscarinic receptors

## Abstract

Basal forebrain cholinergic neurons (BFCNs) densely innervate auditory cortex (ACtx), conveying signals linked to internal brain states and external sensory cues. Acetylcholine (ACh) is known to rapidly modulate cortical circuits through nicotinic ACh receptor (nAChR)-mediated activation of layer 1 inhibitory neurons (L1-INs). However, BFCN terminals are also abundant in deeper layers, where their functional impact has received less attention. Using multi-plex *in situ* labeling across cortical layers and cell types, we found that layer 6 pyramidal neurons (L6-PNs) are highly enriched in diverse transcripts for nAChR subunits and muscarinic ACh receptors (mAChRs). *In vivo* optogenetic activation of BFCN axons revealed persistent modulation of regular spiking units in L2-6 but a rapid phasic activation only in L6. In acute slices, optogenetic activation of BFCN axons elicited fast nAChR-mediated excitatory post-synaptic potentials in L6-PNs, comparable to responses in L1-INs, and slower mAChR-mediated inhibitory responses. These findings identify L1-INs and excitatory L6-PNs as two major hubs for BFCN modulation of cortical circuits. By recruiting distinct receptor mechanisms and circuit motifs in L1 and L6, BFCNs may engage parallel pathways of cholinergic control that couple fast, transient modulation with slower, sustained regulation to shape cortical perception and plasticity.

## Introduction

Acetylcholine (ACh) is a critical neuromodulator that enhances the detection of salient sensory cues (Parikh et al. 2007; Goard and Dan 2009; Pinto et al. 2013; Gritton et al. 2016) and promotes experience-dependent cortical plasticity (Kilgard and Merzenich 1998; Froemke et al. 2007, 2013; Verhoog et al. 2016; Takesian et al. 2018; Yaeger et al. 2019) that supports learning and memory (Bakin and Weinberger 1996; Froemke et al. 2007; Letzkus et al. 2011; Sabec et al. 2018; Guo et al. 2019; Asokan et al. 2023). In the rodent auditory cortex (ACtx), both primary and secondary areas receive dense projections from basal forebrain cholinergic neurons (BFCNs) located in the caudal tail of substantia innominata (SI) and the globus pallidus externa (GPe), with additional projections from more caudal and ventral regions of SI, often labeled as nucleus basalis (Kim et al. 2016; Chavez and Zaborszky 2017). Caudal BFCNs exhibit sound-evoked and behaviorally-gated activation patterns at multiple timescales—from fast, transient bursts to sustained activity—that shape sensory processing and plasticity in a context-dependent manner (Letzkus et al. 2011; Eggermann et al. 2014; Fu et al. 2014; Hangya et al. 2015; Nelson and Mooney 2016; Reimer et al. 2016; Kuchibhotla et al. 2017; Crouse et al. 2020; Robert et al. 2021; Zhu et al. 2023; Kimchi et al. 2024).

The impact of ACh on cortical function depends not only on the timing of ACh release but also on the distribution and subtype diversity of its receptors across the cortical layers. Ionotropic nicotinic ACh receptors (nAChRs) and G protein-coupled muscarinic ACh receptors (mAChRs) exhibit varying affinities, kinetics and downstream intracellular signaling cascades, enabling ACh to exert both phasic and tonic effects on cell excitability, functional synaptic connectivity, and plasticity (Disney and Higley 2020; Sarter and Lustig 2020). While recent studies have largely focused on the influence of ACh on superficial inhibitory microcircuits, less is known about cholinergic modulation within deeper cortical layers.

Cholinergic axons densely innervate deep cortical layers across both sensory and non-sensory cortices (Mechawar et al. 2000; Bloem et al. 2014; Allaway et al. 2020). In ACtx, layer 6 pyramidal neurons (L6-PNs) receive monosynaptic input from BFCNs (Clayton et al. 2021). L6-PNs have recently been recognized as key nodes in regulating cortical output and perceptual behavior, mediating sensory gain control, and shaping cortical representation of sensory stimuli (Olsen et al. 2012; Bortone et al. 2014; Crandall et al. 2015; Guo et al. 2017; Williamson and Polley 2019; Voigts et al. 2020; Clayton et al. 2021). These L6-PNs comprise distinct subpopulations based on their projection targets (Harris and Shepherd 2015). In the ACtx, intratelencephalic L6-PNs communicate with regions such as the neocortex, striatum, and amygdala (Prieto and Winer 1999; Winer and Prieto 2001), small extratelencephalic L6-PNs at the white matter border innervate the outer shell of the inferior colliculus (Schofield 2009; Yudintsev et al. 2021), and corticothalamic (CT) L6-PNs provide feedback to the ipsilateral thalamus (Prieto and Winer 1999; Guo et al. 2017; Clayton et al. 2021). CT L6-PNs also modulate sensory processing within the local circuit through projections onto inhibitory neurons and other deep layer corticofugal PNs (Kim et al. 2014). Despite their privileged position at the interface of sensory input and cortical output, the mechanisms by which ACh influences these L6-PNs remain unresolved.

Here, we mapped ACh receptor heterogeneity across the cortical layers of primary ACtx and found that nAChR and mAChR transcripts are enriched within L6-PNs. Activation of BFCN axons *in vivo* induced both transient and persistent changes in the firing rate of L6 regular spiking (RS) units, in agreement with the co-expression of diverse nAChR and mAChR subtypes. Using an acute slice preparation, we found that BFCN axon stimulation elicited both nAChR-mediated depolarizing and mAChR-mediated hyperpolarizing responses in L6-PNs of ACtx slices. Together, these findings identify L6 of the mouse primary ACtx as a hub for cholinergic modulation and support a mechanism by which ACh may shape cortical output during auditory processing.

## Materials and methods

**Experimental model.** All experiments were carried out in mice generated by crossing ChAT-IRES-Cre(∆neo) males (RRID: IMSR_JAX:031661) with Cdh23 females (RRID: IMSR_JAX:018399), both obtained from the Jackson Laboratory. Mice were group housed and maintained under a 12:12 hr light:dark cycle, with access to food and water ad libitum. Both male and female adult mice (postnatal days 60-90) were used. All procedures were approved by the Institutional Animal Care and Use Committee at Massachusetts Eye and Ear (approval number 2021N000266).

**Surgeries.** Mice were anesthetized with 5% isoflurane in O_2_ and moved to a stereotaxic surgery rig where the mouse was maintained on 2% isoflurane during the procedure. A homeothermic blanket system was used to maintain body temperature at 36.5 °C. After shaving and disinfecting the skin, the dorsal surface of the scalp was retracted, and the periosteum was removed.

For multiplex fluorescence in situ hybridization (FISH) experiments, a burr hole was made in the skull (coordinates: A-P: 2.4; M-L: 2.2, depth: 2.95 mm and 3.15 mm) to target the right medial geniculate body (MGB). A motorized injection system (Nanoject) was used to deliver the retrograde tracer Cholera Toxin subunit-B (CTB) Alexa Fluor-647 (Thermofisher, C34778) at two depths (75 nL per site, 9 nL/min).

For *in vitro* and *in vivo* electrophysiology experiments, a burr hole was made in the skull at 0.8 mm posterior from bregma and 2.7 lateral from the midline, to target the right caudal tail of the cholinergic basal forebrain. At this site, mice were injected with 500 nL of AAV2/5-Ef1a-DIO-ChR2-EYFP (Mass Eye and Ear Viral Vector core, Addgene plasmid #35509), at a depth of 3.45 mm below the pial surface (20 nL/min).

For *in vivo* electrophysiology and BFCNs optogenetic stimulation, the skull surface was prepped with etchant (C&B metabond) and 70% ethanol before affixing a titanium head plate (iMaterialise) to the dorsal surface with dental cement (C&B metabond). A ground wire (AgCl) was implanted over the left occipital cortex. All electrophysiology experiments were performed four weeks after viral injection. At the beginning of all recovery procedures, Buprenex (1 mg/kg) and Meloxicam (5 mg/kg) were administered and following procedures, the animal was transferred to a warm recovery chamber.

**Histology and in situ hybridization.** Five days after CTB Alexa Fluor 647 injections, mice were anesthetized with 5% isoflurane in O_2_, and perfused transcardially with 4% paraformaldehyde (PFA) in phosphate buffer. The brains were removed and post-fixed for 24 h, then transferred to a 30% sucrose solution at 4 ^o^C for cryoprotection. Coronal sections (10 μm) were obtained from the rostral-caudal extent of the primary ACtx of fresh frozen brains. Multiplexed FISH was used to detect expression of nAChR subunit transcripts (β2, α4, and α7) and mAChR (M1, M2, M3, M4) transcripts in fresh frozen tissue sections from primary ACtx (Table 1). Neuronal subtypes were identified by expression of transcripts for the vesicular glutamate transporter 1 (VGluT1, *Slc17a7*) or the vesicular GABA transporter (VGAT, *Slc32a1*). Assays utilized RNAscope probes, reagents, and protocols produced by Advanced Cell Diagnostics (ACD, Hayward, CA) for multiplexed FISH, following ACD protocols and procedures previously described (Ghimire et al. 2020, 2023). Briefly, sections were post-fixed for 60 min in 4% PFA in phosphate buffer, dehydrated in an ascending ethanol series, immersed in ACD Target Retrieval solution for 5 min at 95 ^o^C, incubated for 30 min in Protease 3 solution at 40 ^o^C, followed by probe hybridization (cocktail of all probes) for 2 h at 40 ^o^C. Sequential amplification steps culminated in binding of fluorescent conjugates (T1-Alexa 488, T2-Atto 555, T4-Alexa 750) to probe channels T1, T2, and T4 and counterstaining with DAPI. Sections were imaged, fluorescent tags stripped, then amplification proceeded for probes T5, T6, and T8, followed by imaging for T5—T8, stripping, amplification for probes T9, T10, and T12, then imaging for probes T9—T12. The T3, T7, and T11 channels were not hybridized with any probe, as that channel contained the CTB Alexa 647 signal, serving as a marker of cells labeled by retrograde CTB transport.

**Table 1:** RNAscope reagents and probes for nAChRs, mAChRs, and neuronal class detection.

Cell type	Target	Gene	Cat. No.	Accession No.	Position
All	Nicotinic receptor subunit, α4	*Chrna4*	429,871-T1	NM_015730.5	1129 - 2273
All	Nicotinic receptor subunit, α7	*Chrna7*	465,161-T2	NM_007390.3	175 - 1122
Glutamatergic neurons	Vesicular glutamate transporter 1, VGluT1	*Slc17a7*	416,631-T4	NM_182993.2	464 - 1415
All	Nicotinic receptor subunit, β2	*Chrnb2*	449,231-T5	NM_009602.4	232 - 1805
All	Muscarinic receptor 1	*Chrm1*	495,291-T6	NM_001112697.1	851 - 1994
All	Muscarinic receptor 2	*Chrm2*	495,311-T8	NM_203491.3	940 - 1960
All	Muscarinic receptor 3	*Chrm3*	437,701-T9	NM_033269.4	353 - 1395
All	Muscarinic receptor 4	*Chrm4*	410,581-T10	NM_007699.2	400 - 1330
GABAergic neurons	Vesicular GABA transporter, VGAT	*Slc32a1*	319,191-T12	NM_009508.2	894 - 2037
----	RNAscope HiPlex12 Reagents Kit	----	324,409	----	----

**Multi-fluorescence imaging and cellular phenotyping.** Images of multiplex FISH-reacted sections were obtained using a 20x objective with a Nikon 90i epifluorescence microscope and Hamamatsu Orca 4.0 CCD camera, controlled by Nikon Elements AR software. Each of the three images sets (T1—T4, T5—T8, T9—T12) were imaged in 5 color channels, aligned by DAPI staining, then merged to form a resultant multiplexed image containing the three nAChR and four mAChR channels, plus CTB, and DAPI. Images were imported into HALO pathology software (Indica labs, Albuquerque, NM) for analysis.

Transcript density was obtained by counting individual transcripts for each nAChR and mAChR target and quantified by cortical layer. Cells expressing the transcripts of each nAChR and mAChR target were tallied by cortical layer and neuronal class, based on co-expression with VGluT1 (glutamatergic), VGAT (GABAergic) and CTB. Cells that contained 5 or more transcripts met the threshold for tagging as positively labeled for a given probe target. Cellular phenotypes were identified based on co-expression patterns of the nAChR and mAChR targets and were tallied by neuronal subtype and cortical layer.

**Single unit recordings during optogenetic stimulation in head-fixed mice.** Animals were briefly anesthetized with isoflurane (5% in O_2_ for induction, 2% during the procedure) while a small 1 mm x 1 mm craniotomy was made along the caudal end of the right temporal ridge to expose the primary ACtx (1.5 mm rostral from lambda). A small chamber was built around the craniotomy with UV-cured cement and filled with lubricating ointment (Paralub Vet Ointment). At the conclusion of each recording, the chamber was flushed, filled with new ointment, and capped with UV-cured cement.

A 64-channel silicon probe (H3, Cambridge Neurotech) was slowly advanced (100 mm/s) into the primary ACtx perpendicular to the pial surface until the tip of the electrode was 1.3 to 1.4 mm below the cortical surface to cover all layers of primary ACtx. The brain was allowed to settle for at least 15 min before recordings began. On the day of the first recording, multiple penetrations were made to identify the tonotopic reversal which represents the rostral border of the primary ACtx (Guo et al. 2012). Raw neural data was digitized at 32-bit, 24.4 kHz and stored in binary format (PZ5 Neurodigitizer, RZ2 BioAmp Processor, RS4 Data Streamer; Tucker-Davis Technologies). To eliminate artifacts, the common mode signal (channel-averaged neural traces) was subtracted from all channels in the brain. Signals were notch filtered at 60 Hz and band-pass filtered (300-3000 Hz, second order Butterworth filter).

To calculate local field potentials (LFP), raw signals were first notch filtered at 60 Hz and downsampled to 1 kHz. The current source density (CSD) was calculated as the second spatial derivative of the LFP signal. To eliminate potential artifacts introduced by impedance mismatching between channels, signals were spatially smoothed along the channels with a triangle filter (5-point Hanning window). Two CSD signatures were used to identify layer 4 (L4) in accordance with prior studies: (i) a brief current sink approximately 10 ms after the onset of a broadband noise burst (50 ms duration, 70 dB SPL, 50 trials) was used to define the lower border of L4 (Kaur et al. 2005), and (ii) a tri-phasic CSD pattern (sink-source-sink from upper to lower channels) between 20 to 50 ms defined the upper boundary of L4 at the transition between the upper sink and the source (Müller and Mitzdorf 1984; Metherate et al. 2005; Guo et al. 2017; Clayton et al. 2021). Single unit clusters were obtained using Kilosort (Pachitariu et al. 2016), and single unit isolation was based on the presence of both a refractory period within the interspike interval histogram, and an isolation distance (> 10) indicating that single unit clusters were separated from the surrounding noise (Schmitzer-Torbert et al. 2005; Clayton et al. 2021).

Optogenetic activation of BFCN axon terminals was achieved using an optic fiber/ferrule assembly (0.2 mm diameter, 0.22 NA Doric) coupled to a 473 nm diode laser for ChR2 activation (Omnicron LuxX) at 2.55 mW/mm^2^.

**In vitro electrophysiology.** Four weeks after viral delivery of ChR2, mice were anesthetized with 5% isoflurane in O_2_ followed by intraperitoneal administration of Fatal Plus (0.22 mL/kg). Immediately after induction, mice were transcardially perfused with ice-cold slicing artificial cerebrospinal fluid (ACSF) containing (in mM): 160 sucrose, 28 NaHCO_3_, 2.5 KCl, 1.25 NaH_2_PO_4_, 7.25 glucose, 20 HEPES, 3 Na-pyruvate, 3 Na-ascorbate, 7.5 MgCl_2_, 1 CaCl_2_. Brains were rapidly removed, and thalamocortical slices (300 μm) containing the right primary ACtx were obtained by slicing at an angle of 15° from the horizontal plane (Cruikshank et al. 2002) using a vibrating microtome (Leica Microsystems; VT1200S).

Slices were incubated for 30 min at 35 °C in recovery ACSF containing (in mM): 92 NaCl, 28.5 NaHCO_3_, 2.5 KCl, 1.2 NaH_2_PO_4_, 25 glucose, 20 HEPES, 3 Na-pyruvate, 5 Na-ascorbate, 4 MgCl_2_, 2 CaCl_2_. After recovery, slices were transferred to recording ACSF, containing (in mM): 125 NaCl, 2.5 KCl, 1.25 NaH_2_PO_4_, 25 NaHCO_3_, 25 glucose, 1 MgCl_2_, 2 CaCl_2_. During recordings, slices were continuously superfused with oxygenated recording ACSF (95% O_2_/5% CO_2_) and maintained at 31 °C to 33 °C.

Recording electrodes (3-5 MΩ) were pulled from borosilicate glass capillaries using a micropipette puller (P-97, Sutter Instrument) and filled with current-clamp internal solution containing (in mM): 5 KCl, 127.5 K-gluconate, 10 HEPES, 2 MgCl_2_, 0.6 EGTA, 2 Mg-ATP, 0.3 Na-GTP, 5 Na_2_-phosphocreatine; pH 7.2, adjusted with KOH (Takesian et al. 2013). Cells with series resistance < 30 MΩ were included for analysis, and resistance was compensated up to 60%. Data were acquired at 10 kHz using a Multiclamp 700B amplifier (Molecular Devices), low-pass filtered at 3 kHz, and digitized via a NI-USB-6343 (National Instruments). Custom-designed MATLAB 2018 software was used for data acquisition (Bernardo Sabatini's laboratory). All recordings were conducted using a motorized upright microscope (Scientifica, SliceScope Pro 1000) coupled to a CCD camera (Hamamatsu Photonics, Orca Flash 4.0) and PatchStar micromanipulators (Scientifica).

Optogenetic stimulation of BFCN axons in the primary ACtx was performed using a wide-field 470 nm LED pulse (CoolLED, pE-100) delivered through the microscope (5 ms, ~ 14 mW/mm^2^). BFCN axons-evoked excitatory or inhibitory postsynaptic potentials (E/IPSPs) were recorded from visually identified layer 1 inhibitory neurons (L1-INs) or layer 6 pyramidal neurons (L6-PNs) under IR-DIC optics, in the presence of 20 μM DNQX and 50 μM AP5 to prevent glutamatergic transmission. The cholinergic identity of recorded responses was further confirmed by sequential bath application of the nAChR antagonists methyllycaconitine (MLA, 10 μM) and dihydro-β-erythroidine hydrobromide (DhβE, 10 μM), and the mAChR antagonist atropine (10 μM), depending on the response profile observed in each recorded neuron.

**Quantification and statistical analysis.** For single unit recordings, analyses were performed using custom routines in MATLAB 2021 (MathWorks). Units were classified based on the ratio of the mean trough-to-peak interval as regular spiking (RS, > 0.6 ms) or fast spiking (FS, < 0.5 ms). For each unit, the average of 50 trials was z-scored and plotted as neurograms (Fig. 4D). For the absolute average z-scored values (Fig. 4E), calculations were performed for the baseline, onset, and persistent time periods as follows: for baseline, time bins of the z-scored response were shuffled 1000 times and for each shuffle, three consecutive bins were chosen at random, their absolute z-scores averaged to obtain 1000 values that were subsequently averaged to obtain a single baseline value per unit; for onset, the absolute z-scores of the three bins corresponding to this time domain were averaged; for persistent, the absolute z-scores of the time bins between 150 and 600 ms were averaged, corresponding to a time window during which muscarinic effects were observed *in vitro*. Statistical comparisons between the three time periods during the *in vivo* experiments (Fig. 4E, Table S4) were made using mixed-effects one-way ANOVA, nested within mice. For Fig. 4F values for “L2-5” and “L6” correspond to the Onset period in Fig. 4E, and shuffled control values were obtained for each unit by shuffling the time bins of the entire neurogram 1000 times and averaging three bins.

For *in vitro* whole-cell recordings, excitatory, and inhibitory postsynaptic potential (E/IPSP) amplitudes and intrinsic properties were quantified using Clampfit 10.7 (Molecular Devices) and custom-designed MATLAB 2018 routines. Intrinsic passive and active neuronal properties were determined by applying 1 s current steps from −190 pA to 200 pA in increments of 10 pA while recording in current-clamp whole-cell configuration. Membrane capacitance (C_m_), input resistance (R_input_), and membrane tau were measured from a 50 pA hyperpolarizing pulse. The resting membrane potential was defined as the mean initial potential across sweeps before current injection. Rheobase was defined as the minimal depolarizing current required to elicit an action potential. Action potential threshold was defined as the membrane potential at which dV/dt reached 7.5% of the maximum dV/dt before the action potential peak. The latency to the first action potential was calculated as the time between the stimulus onset and the action potential threshold. The half-width was calculated as the average width of all action potentials during the 200 pA step, measured at 50% of the membrane potential between the action potential threshold and peak. The action potential height was measured as the difference between the action potential peak and trough, also averaged across all the action potentials during the 200 pA step. Statistical analyses were performed in GraphPad Prism 10. Data are reported as mean ± SEM, unless otherwise indicated.

## Results

### Diverse nicotinic and muscarinic ACh receptor subtypes are expressed in L6 of the primary ACtx

**Fig. 1:**
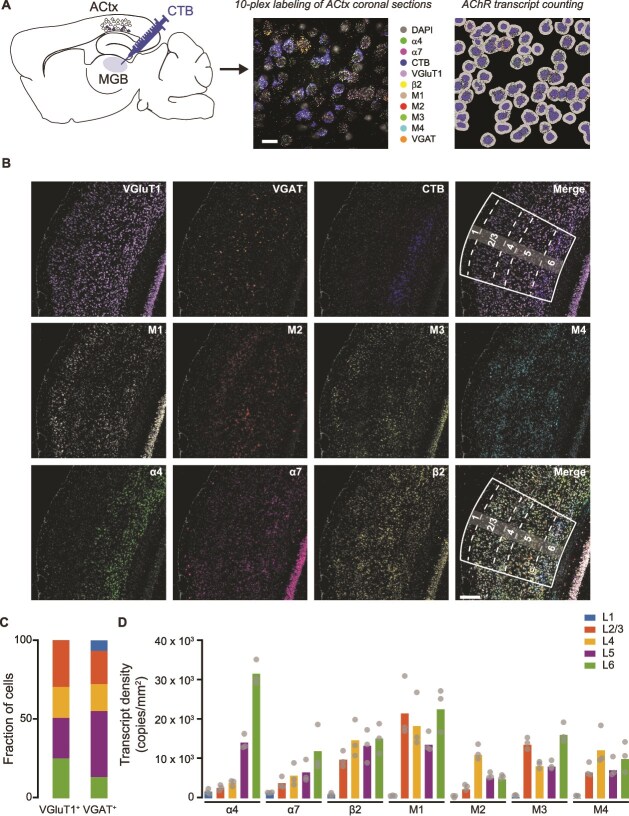
**nAChR and mAChR transcripts are expressed across all layers in primary ACtx. A)** Schematic of the multiplex FISH strategy. **Left**: mice (*n* = 3) were injected with CTB-Alexa fluor 647 in the right MGB to label CT-PNs. **Middle**: 10-plex FISH localized mRNA transcripts encoding nAChRs and mAChRs within excitatory (VGluT1^+^) and inhibitory (VGAT^+^) neurons. **Right:** individual AChR transcripts were counted within the cytoplasm of excitatory and inhibitory neurons. Scale bar: 20 μm. **B)** Images of primary ACtx sections showing multiplex FISH labeling of mRNA transcripts encoding VGluT1^+^ and VGAT^+^, and mRNA transcripts encoding nAChR subunits (α4, α7, β2) and mAChRs (M1-4). Scale bar: 200 μm. Laminar boundaries are indicated by dashed lines. **C)** Distribution of excitatory (VGluT1^+^, *n* = 2947) and inhibitory (VGAT^+^, *n* = 410) neurons across ACtx layers. **D)** Quantification of the density of mRNA transcripts encoding nAChRs and mAChRs across ACtx layers.

Cholinergic axons innervate all layers of sensory cortex, with the highest densities observed in the superficial and deep layers (Mechawar et al. 2000; Allaway et al. 2020). To investigate the laminar specificity of cholinergic modulation in ACtx, we first quantified the distribution of mAChR and nAChR transcripts across cortical layers and cell types in the primary ACtx. CT neurons within primary ACtx were labeled by injecting the retrograde tracer CTB into the MGB, and primarily concentrated in L6 (Prieto and Winer 1999; Guo et al. 2017; Williamson and Polley 2019; Clayton et al. 2021) (Fig.  1A).

We performed multiplex FISH to localize mRNA transcripts encoding nAChRs and mAChRs within excitatory (vesicular glutamate transporter 1; VGluT1^+^) and inhibitory (vesicular GABAergic transporter; VGAT^+^) neurons (Fig.  1A to C). Transcripts for the α4, α7, and β2 nAChR subunits and M1-4 mAChR receptors were widely expressed across the primary ACtx (Fig.  1B and D; Table S1). Notably, we observed enriched expression of both nAChRs and mAChR subunit transcripts within L6, particularly α4 nAChR subunit transcripts, consistent with findings in rat ACtx (Ghimire et al. 2020).

**Fig. 2:**
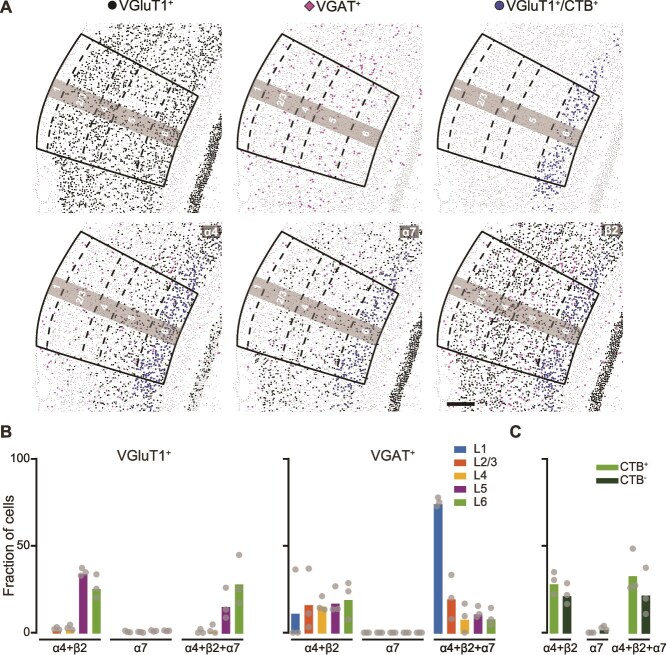
**nAChR subunit transcripts are enriched in L6 of primary ACtx. A) Top:** plots of cells identified as excitatory neurons (VGluT1^+^), inhibitory neurons (VGAT^+^), and CT-PNs (VGluT1^+^/CTB^+^). **Bottom:** cells containing transcripts for each nAChR subunit in VGluT1^+^, VGAT^+^ and VGluT1^+^/CTB^+^ cells. Laminar boundaries are indicated by dashed lines. Scale bar: 200 μm. **B)** Fraction of excitatory (VGluT1^+^, left) and inhibitory (VGAT^+^, right) neurons expressing subunit transcript combinations to putatively form heteromeric α4β2 only, homomeric α7 nAChRs only, or both α4β2 and α7 nAChRs across ACtx layers. **C)** Fraction of CT L6-PNs (CTB^+^) or non-CT L6-PNs (CTB^−^) expressing nAChR subunit transcripts.

The predominant nAChR subtypes in cortex are the homomeric α-bungarotoxin (α-Bgtx)-sensitive nAChR, composed of five α7 subunits, and the heteromeric α-Bgtx-insensitive nAChR, composed of α4 and β2 subunits (Radnikow and Feldmeyer 2018; Zoli et al. 2018). We quantified the presence of transcripts encoding these subunits within VGAT^+^ and VGluT1^+^ populations (Fig.  2A). Cortical L1 inhibitory neurons (L1-INs) are a major target for ACh across neocortical areas, where signaling occurs primarily through nAChRs (Letzkus et al. 2011; Takesian et al. 2018). We found that the majority (75.17 ± 1.19%) of VGAT^+^ L1-INs expressed mRNA transcripts encoding the α7, α4, and β2 subunits, likely producing homomeric α7 and heteromeric α4β2 nAChRs (Fig.  2B; Table S2). Across other layers, no more than 25% of VGAT^+^ neurons expressed transcripts encoding putative α7 and α4β2 nAChRs (Fig.  2B; Table S2). Among the VGluT1^+^ neuronal populations, those in L5 and L6 showed the highest fraction of neurons expressing transcripts encoding both α7 and α4β2 nAChRs or only the α4β2 nAChR (Fig.  2B; Table S2). Within the CTB^+^ population of CT L6-PNs, the ratio of neurons expressing these nAChR subunit transcripts was modestly elevated as compared to the CTB^−^ population (Fig.  2C; Table S3). Consistent with observations in rat ACtx (Ghimire et al. 2020), we also found a subpopulation of VGAT^+^ cells spanning L2-5 that might express α7β2 nAChRs.

**Fig. 3:**
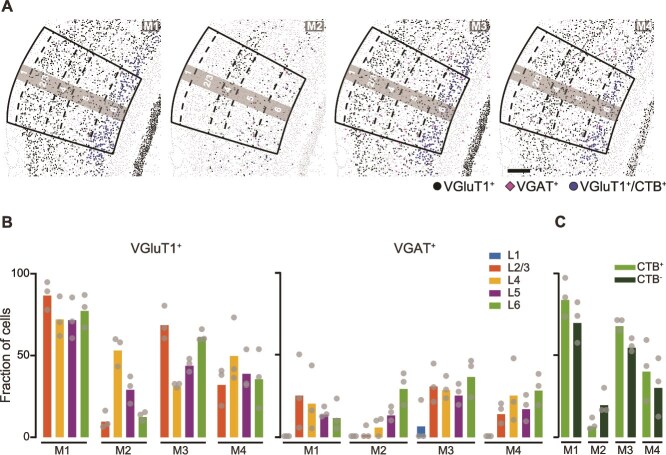
**Robust expression of mAChR transcripts across primary ACtx. A)** Plots excitatory neurons (VGluT1+), inhibitory neurons (VGAT+), and CT-PNs (VGluT1+/CTB+) containing mAChR transcripts. Laminar boundaries are indicated by dashed lines. Scale bar: 200 μm. **B)** Fraction of excitatory (VGluT1^+^, left) and inhibitory (VGAT^+^, right) neurons expressing mAChR subtype transcripts (M1-4) across primary ACtx layers. **C)** Fraction of CT L6-PNs (CTB^+^) or non-CT L6-PNs (CTB^−^) expressing mAChR transcripts.

We next analyzed the expression of mAChR subtype (M1-4) transcripts. A higher proportion of VGAT^+^ inhibitory neurons in L6 expressed M2, M3, and M4 receptor transcripts as compared to inhibitory neurons within the other cortical layers. The majority of VGluT1^+^ neurons across all layers of ACtx expressed mRNAs for the M1 receptor (Fig.  3A and B; Table S2). Within the CTB^+^ population of CT L6-PNs, a greater proportion of neurons expressed M1, M3, and M4 receptor transcripts, whereas more CTB^−^ neurons expressed M2 mRNAs (Fig.  3C; Table S3). Together, these transcriptomic data reveal that both excitatory and inhibitory neurons in L6 express diverse nAChR and mAChR subtypes, highlighting L6 as a key target for ACh modulation.

### Cholinergic input modulates spiking in L6 of the primary ACtx

**Fig. 4:**
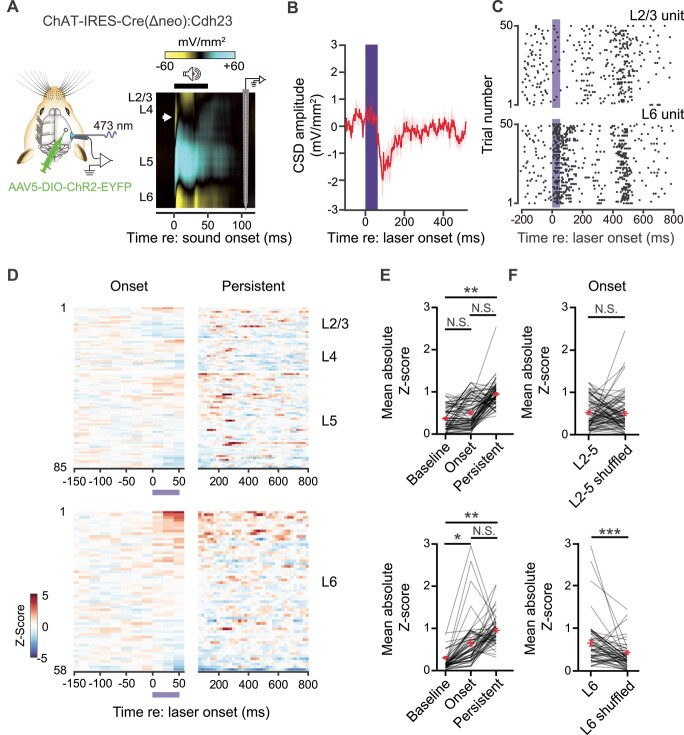
**BFCNs modulate L6 primary ACtx neurons in vivo. A)** Schematic of the experimental strategy for *in vivo* single unit recordings. Cre-dependent ChR2 was injected into the caudal tail of the cholinergic basal forebrain of ChAT-IRES-Cre(Δneo):Cdh23 mice (*n* = 4 mice). Neural activity was recorded with a 64-channel linear silicon probe. BFCN axons in primary ACtx were stimulated with an optic fiber coupled to a 473 nm diode laser (**left**). Extracellular recordings were performed through L2-6 in primary ACtx. The CSD of neural activity in response to a 50 ms noiseburst was used to approximate depth for each recorded unit (**right**). The white arrow indicates the early current sink elicited by the stimulus, demarcating the L4/5 boundary. **B)** A brief laser pulse (50 ms, 30 mW) was used to determine the presence of the L2/3 sink in the CSD evoked by cholinergic activation (Guo et al. 2019). **C)** Raster plots of spiking activity across trials for representative units in L2/3 (**top**) and L6 (**bottom**). **D)** Neurograms representing the Z-scored responses of RS units to a laser pulse (50 ms, 30 mW) used to activate the BFCN axons in ACtx. For each layer, units are sorted by mean Z-score responses during the laser stimulus. **E)** Mean absolute Z-score values for units in L2-5 (**top**) or in L6 (**bottom**) calculated before the laser stimulation (baseline; L2-5: 0.37 ± 0.03; L6: 0.29 ± 0.02), during the 50 ms laser stimulation (onset; L2-5: 0.51 ± 0.04; L6: 0.65 ± 0.07) and during the period between 150 ms and 600 ms after the laser onset (persistent; L2-5: 0.95 ± 0.03; L6: 0.96 ± 0.04). Mixed-effects one-way ANOVA with Tukey’s post-hoc comparison (F_2,9_ = 9.728; *P* = 0.0078) for L2-5 (**top**): Baseline vs. onset (*P* = 0.12); baseline vs. persistent (*P* = 0.006); onset vs. persistent (*P* = 0.19). Mixed-effects one-way ANOVA with Tukey’s post-hoc comparison (F_2,9_ = 9.604; *P* = 0.0059) for L6 (**bottom**): Baseline vs. onset (*P* = 0.04); baseline vs. persistent (*P* = 0.005); onset vs. persistent (*P* = 0.33). **F)** Mean absolute Z-score during the onset time window compared to a shuffled control for L2-5 (**top**; onset: 0.51 ± 0.04 and control: 0.50 ± 0.05) and L6 (**bottom**; onset: 0.65 ± 0.07 and control: 0.44 ± 0.04). Wilcoxon test for L2-5 vs. L2-5 shuffled (*P* = 0.66) and L6 vs. L6 shuffled (*P* = 0.0001).

To probe the functional impact of cholinergic inputs on ACtx neurons across the cortical layers, we selectively expressed channelrhodopsin-2 (ChR2) in BFCNs by injecting AAV2/5-Ef1a-ChR2-EYFP into the caudal tail of the cholinergic basal forebrain of ChAT-IRES-Cre(Δneo):Cdh23 mice (Fig. 4A), which offer excellent hearing into adulthood and selective expression of Cre-recombinase in cholinergic neurons (Robert et al. 2021). Four weeks post-injection, we performed translaminar extracellular recordings from primary ACtx of awake, head-fixed mice while optogenetically stimulating BFCN axons (Fig. 4A).

Cortical layer boundaries were identified using CSD profiles evoked by a 50 ms broadband noise burst, with a characteristic sink demarcating the boundary between L4 and L5 (Fig. 4A). BFCN axon activation elicited CSD responses in L2/3, confirming that activation of cholinergic axons elicited local network activity in the primary ACtx (Fig. 4B) (Guo et al. 2019). However, cholinergic axons innervate all layers of the cortex (Mechawar et al. 2000; Bloem et al. 2014; Allaway et al. 2020), imposing a challenge to isolate the contributors of the BFCN-evoked CSD.

We then isolated single units in L2-6 with RS waveforms to investigate laminar differences in cholinergic modulation of putative PNs. Although cholinergic inputs to the neocortex are generally viewed as modulatory and therefore unlikely to directly elicit spikes, we observed that some units exhibited elevated spiking hundreds of milliseconds after BFCN axon stimulation while other units exhibited robust spiking shortly following BFCN activation (Fig. 4C). We quantified these effects by contrasting the absolute mean z-scored activity of each unit shortly following BFCN activation (Onset; 0-50 ms post-laser onset) to the spiking activity at longer delays after BFCN activation that may be mediated by polysynaptic intracortical circuits (150 ms to 600 ms post-laser onset; Fig. 4D). BFCN axon stimulation elicited a weak persistent elevation in spiking in all layers, but time-locked BFCN-evoked onset responses were only observed in L6 (Fig. 4E, Table S4). To confirm that BFCN axon activation elicited temporally coherent onset responses, we compared each unit’s activity during Onset to a time-shuffled control of its spiking activity throughout the post-stimulus period (Fig. 4F). This analysis confirmed that BCFN axon stimulation could act as a “driver” for L6 putative PNs, eliciting short-latency spiking that could arise from the abundance of nAChRs in deep layer excitatory neurons. When considered alongside the temporally dispersed persistent changes in spiking, these findings demonstrate that BFCN inputs modulate L6 RS units in primary ACtx and may act through both fast and slow receptor mechanisms to produce layer-specific cholinergic modulation.

### L6-PNs in the primary ACtx exhibit mAChR- and nAChR-mediated responses in vitro

**Fig. 5:**
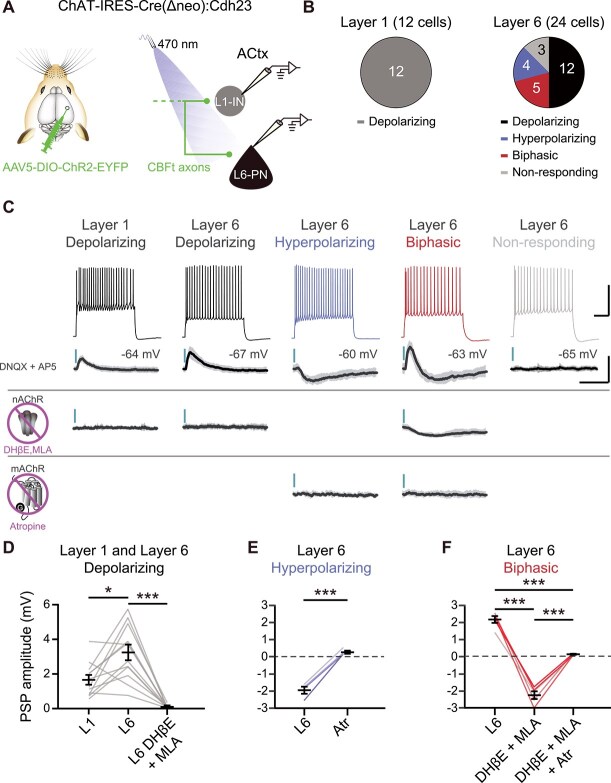
**BFCN axon stimulation evokes nAChR- and mAChR-mediated postsynaptic responses in L6-PNs neurons within primary ACtx. A)** Schematic of the experimental strategy. Cre-dependent ChR2 was injected into the caudal tail of the cholinergic basal forebrain in ChAT-IRES-Cre(∆neo):Cdh23 mice (*n* = 6 mice). BFCN axons in ACtx were optically stimulated (470 nm LED, 14 mW/mm^2^, 5 ms) and whole-cell current clamp recordings were obtained from L1-INs and L6-PNs. **B)** Pie chart summarizing BFCN-evoked PSPs. All L1-INs exhibited monosynaptic depolarizing PSPs, whereas L6-PNs displayed monosynaptic depolarizing, hyperpolarizing and biphasic PSPs. **C) Top:** example recordings in L1-INs and L6-PNs characterized by spiking patterns in response to intrinsic current pulses. Scale bar: 50 mV, 250 ms. **Middle**: BFCN-evoked PSPs (mean ± SD of 10 trials) recorded in the presence of AMPA and NMDA receptor blockers (DNQX, 20 μM; AP5, 50 μM). **Bottom:** PSPs were abolished by nAChR antagonists DHβE (10 μM) and MLA (10 μM) and by the mAChR antagonist atropine (10 μM). Scale bar: 5 mV, 500 ms. **D)** Depolarizing PSPs in L6-PNs (3.25 ± 0.45 mV) were significantly larger than those in L1-INs from the same slices (1.66 ± 0.28 mV) and were eliminated by nAChR antagonists DHβE and MLA (0.09 ± 0.03 mV). Wilcoxon test L1 vs L6: *P* = 0.012; Wilcoxon test L6 vs L6 DHβE + MLA: *P* = 0.0005. **E)** Hyperpolarizing PSPs in L6-PNs (−2.05 ± 0.21 mV) were abolished by atropine (Atr, 0.18 ± 0.09 mV). Paired *t*-test L6 vs Atr: *P* = 0.001. **F)** Biphasic PSPs in L6-PNs (2.15 ± 0.20 mV) were sequentially eliminated by DHβE + MLA (−2.27 ± 0.23 mV) and atropine (Atr, 0.12 ± 0.03 mV). RM-ANOVA with post-hoc Fisher’s comparison (F_2,8_ = 139.60, *P* < 0.0001): L6 vs L6 DHβE + MLA (*P* = 0.0002); L6 vs L6 DHβE + MLA + Atr (*P* = 0.0004); L6 DHβE + MLA vs L6 DHβE + MLA + Atr (*P* = 0.0005).

To resolve the receptor-specific contributions underlying fast and sustained cholinergic effects in L6 and to confirm that BFCNs have monosynaptic effects on L6-PNs, we turned to an *in vitro* approach. We performed whole-cell patch-clamp recordings from L6-PNs in acute primary ACtx slices during optogenetic stimulation of BFCN axons (Fig.  5A). Glutamatergic transmission was blocked with AMPA and NMDA receptor antagonists to isolate cholinergic effects.

BFCN axon activation evoked heterogeneous postsynaptic responses in L6-PNs (Fig.  5B). Among recorded neurons, 50% exhibited depolarizing postsynaptic potentials that were eliminated by nAChR antagonists MLA and DHβE (Fig.  5B and C). These depolarizing responses were significantly larger than those recorded in L1-INs recorded within the same slices (Fig.  5B and C). A subset (~20%) of L6-PNs showed exclusively hyperpolarizing PSPs that were abolished by the mAChR antagonist atropine (Fig.  5B and D). Additionally, 16% of L6-PNs exhibited biphasic responses consisting of an initial depolarization followed by a sustained hyperpolarization, which were sequentially abolished by nAChR and mAChR blockade, respectively (Fig.  5B and D). Notably, BFCN axon stimulation-evoked response types were not correlated with differences in intrinsic membrane or action potential properties among L6-PNs (Table S5).

Together, these findings indicate that L6-PNs are key nodes of direct cholinergic input mediated both by fast-acting ionotropic nAChRs and slower-acting metabotropic mAChRs. The combination of depolarizing and hyperpolarizing responses indicates that cholinergic input exerts temporally precise and functionally complex effects on L6 circuitry within the primary ACtx.

## Discussion

Decades of research have established ACh as a key modulator of cortical processing and a driver of plasticity, yet the precise cellular and circuit mechanisms through which it operates remain incompletely understood (Picciotto et al. 2012; Obermayer et al. 2017). Although prior studies have largely focused on superficial cortical layers (Letzkus et al. 2011; Arroyo et al. 2012; Poorthuis et al. 2018; Takesian et al. 2018), our data show that L6-PNs, including CT L6-PNs, are robustly modulated by cholinergic input from the BFCNs.

Neocortical cholinergic signaling has been traditionally characterized as slow and volume-mediated, but a revised model has emerged in which both fast and slow ACh-mediated transmission co-exist to shape cortical states and behavior across timescales (Sarter et al. 2009; Disney and Higley 2020; Sarter and Lustig 2020). The spatiotemporal effects of cholinergic inputs are determined by the receptor subtypes involved (Higley and Picciotto 2014), with ionotropic nAChRs mediating fast, synaptic-like excitatory transmission (Albuquerque et al. 2009) and G-protein coupled mAChRs inducing delayed and prolonged excitatory and inhibitory effects (Thiele 2013). Using transcriptomic profiling and functional recordings, we show that L6-PNs express a broad array of both nAChRs and mAChRs. Notably, L6-PNs are enriched for RNA transcripts of α7, α4, and β2 subunits, likely producing α7 and α4β2 nAChRs—the two most prevalent nAChRs in cortex (Zoli et al. 2018; Ghimire et al. 2020). Indeed, ~ 50% of the L6-PNs express these transcripts, a higher fraction than any other cortical excitatory population in our study (Fig.  2). These L6-PNs also robustly express transcripts encoding M1-M4 mAChRs (Fig.  3) that primarily signal via G_q/11_ (M1 and M3) or G_i/o_ (M2 and M4) pathways (Thiele 2013). The downstream effects of these G-protein coupled mAChRs vary by cell type and depend on proximity to signaling molecules and ion channels, resulting in either depolarization or hyperpolarization (Thiele 2013). A striking example is the M1 receptor, which can induce either closure of K^+^ channels to excite neurons (Womble and Moises 1992; Ghamari-Langroudi and Bourque 2004; Carr and Surmeier 2007; Giessel and Sabatini 2010) or opening of SK Ca^2+^-activated K^+^ channels to inhibit them (Brombas et al. 2014). Thus, receptor expression alone cannot predict postsynaptic effects of cholinergic modulation.

The cholinergic receptor diversity in L6-PNs may underlie the heterogenous responses to BFCN axon stimulation observed in vitro (Fig.  5)—ranging from fast, phasic depolarization via nAChRs to delayed hyperpolarization via mAChRs. These dual responses were even observed within single neurons, highlighting a convergence of receptor signaling that may enable flexible, time-dependent modulation of excitability. Consistent with this, *in vivo* single unit recordings revealed that BFCN axon activation can bidirectionally modulate RS L6 units in primary ACtx on distinct timescales, reflecting the co-engagement of fast and slow cholinergic mechanisms (Fig. 4). Some RS L6 units showed phasic, time-locked spiking, suggesting that ACh can act as a rapid, synaptic-like “driver” via nAChRs, paralleling mechanisms observed in L1-INs. In contrast, sustained cholinergic effects observed across layers, including L6, may be mediated by mAChRs. These results support a growing model that cortical ACh signaling in L6 operates through temporally and functionally distinct modes that interact to support dynamic cortical states.

The co-expression of nAChRs and mAChRs in L6-PNs may enable activity-dependent modulation by ACh. For example, ACh transiently excites neurogliaform neurons in superficial cortex via nAChRs at rest, but inhibits these neurons for long time periods via mAChRs during active firing states (Brombas et al. 2014). Analogously, L6-PNs may show distinct cholinergic responses depending on their membrane potential or recent activity history, positioning ACh as a context-dependent modulator of deep-layer excitability.

The functional impact of cholinergic axons is also shaped by an interaction between presynaptic firing patterns and postsynaptic receptor localization (Laszlovszky et al. 2020; Schlingloff et al. 2025). Cholinergic neurons exhibit a range of firing dynamics—from single spikes triggered by aversive events (Hangya et al. 2015) to graded, learning-related responses to sensory cues (Kuchibhotla et al. 2017; Guo et al. 2019; Crouse et al. 2020; Robert et al. 2021). These firing patterns could influence the accumulation of ACh, determining which receptor subtypes are activated. High-frequency firing may produce sustained, elevated ACh levels that could recruit lower-affinity extrasynaptic receptors and mediate modulatory effects over extended timescales. Conversely, lower ACh concentrations could preferentially activate high-affinity, synaptic nAChRs, and mAChRs (Hay et al. 2016; Yang et al. 2020), producing phasic postsynaptic responses. In somatosensory cortex, for example, low concentrations of ACh modulate all recorded L6-PNs via mAChRs, whereas high concentrations selectivity activate CT L6-PNs via α4β2 nAChRs (Qi et al. 2025). Thus, the temporal dynamics of ACh release may selectively engage specific L6-PNs via distinct receptor subtypes, biasing cortical output toward functionally distinct circuit pathways.

Cholinergic modulation of CT L6-PNs may provide a mechanism for linking arousal state to cortical circuit dynamics, thereby enabling flexible auditory processing. In the ACtx, cholinergic signaling is tightly linked to locomotion, reinforcement learning, arousal, and the perceptual salience of ambient sounds (McGinley et al. 2015; Nelson and Mooney 2016; Reimer et al. 2016; Guo et al. 2019; Robert et al. 2021). CT L6-PNs in ACtx are selectively modulated by motor-related inputs that likely originate from BFCNs (Clayton et al. 2021), are enriched in both nAChRs and mAChRs (Figs.  2 and 3), and are therefore ideally positioned to translate state-dependent cholinergic signals into thalamic feedback control. Across cortical areas, ACh depolarizes CT L6-PNs via nAChRs and mAChR activation, whereas neighboring corticocortical neurons are less responsive (Kassam et al. 2008; Sundberg et al. 2018; Yang et al. 2020; Qi et al. 2025). In addition to receiving cholinergic input, CT L6-PNs integrate top-down inputs from diverse brain regions, including higher-order cortical areas (Vélez-Fort et al. 2014).

CT L6-PNs project back to the thalamus (Prieto and Winer 1999; Harris and Shepherd 2015) forming a feedback loop that dynamically regulates thalamic gain during sound processing (Crandall et al. 2015; Guo et al. 2017; Clayton et al. 2021). The timing of CT L6-PN spikes relative to auditory stimuli determines whether thalamic activity is enhanced or suppressed, effectively switching between sensory processing modes that optimize either the detection or discrimination of sounds (Guo et al. 2017). In addition to this thalamic feedback, CT L6-PNs influence local circuit gain by modulating the activity of neighboring interneurons (Bortone et al. 2014; Kim et al. 2014; Guo et al. 2017), and L5-PNs (Kim et al. 2014). Notably, L5 contains corticofugal neurons that project to the dorsal striatum and guide auditory decision-making (Znamenskiy and Zador 2013). The striatum, in turn, is densely interconnected with the GPe (Hegeman et al. 2016), which also receives direct input from deep layers of the ACtx (Ferenczi et al. 2025). Together, these findings suggest that BFCNs located in the GPe may participate in a loop linking cholinergic modulation to thalamocortical feedback and local circuit gain modulation, with CT L6-PNs serving as a central node for context-dependent auditory processing.

Cholinergic inputs to the ACtx not only modulate moment-to-moment encoding of the sensory environment but promote long-term plasticity (Kilgard and Merzenich 1998; Froemke et al. 2007, 2013; Takesian et al. 2018; Guo et al. 2019). These effects are layer-specific and depend on receptor subtype, subcellular localization, and postsynaptic neuron identity (Couey et al. 2007; Poorthuis et al. 2013; Verhoog et al. 2016). In the prefrontal cortex, activation of β2-containing nAChRs on L6-PNs enhances synaptic plasticity (Verhoog et al. 2016). ACh gives rise to dendritic plateau potentials in L5-PNs of the somatosensory cortex (Williams and Fletcher 2019), which are associated with plasticity (Gambino et al. 2014). Moreover, cholinergic modulation of diverse interneuron subtypes across layers can influence cortical plasticity through various inhibitory circuit motifs (Tremblay et al. 2016). Future studies should investigate how ACh regulates long-term plasticity in deep-layer ACtx circuits, particularly under behaviorally relevant conditions.

Our findings suggest that cholinergic signaling in the neocortex is highly circuit-specific, operating through mechanisms shaped by the distribution of receptor subtypes across cell types and cortical layers (Obermayer et al. 2017). This precision reflects the functional topography of the cholinergic basal forebrain, where anterior regions such as the horizontal limb of the diagonal band (HDB) are preferentially engaged by behavioral outcomes, whereas caudal regions, including the GPe and SI, preferentially respond to salient sensory stimuli and aversive events (Robert et al. 2021). Projections from these nuclei exhibit laminar specificity: in somatosensory cortex, HDB and rostral SI axons target superficial layers (e.g. L1), whereas caudal SI and nucleus basalis axons preferentially innervate deeper layers (Allaway et al. 2020). A similar laminar specificity has been observed in the prefrontal cortex (Bloem et al. 2014), suggesting parallel cholinergic pathways that modulate distinct microcircuits.

This spatially-specific organization of cholinergic modulation across the cortical laminae supports a model in which ACh release is not uniform but may dynamically modulate separate cortical circuits in a task-dependent manner. Notably, CT L6-PNs robustly recruit parvalbumin-expressing (PV) interneurons (West et al. 2006; Frandolig et al. 2019), while L1-INs inhibit PV cells (Letzkus et al. 2011; Pi et al. 2013; Abs et al. 2018; Takesian et al. 2018; Cohen-Kashi Malina et al. 2021; Hartung et al. 2024), revealing a complex, multilayered regulatory mechanism. A subpopulation of cortical L6-PNs may also send projections to L1-INs, suggesting interlaminar communication (Ledderose et al. 2023). Together, these observations support a model in which cholinergic inputs to L1 and L6 engage distinct, and potentially competing, microcircuits that modulate sensory processing across behavioral states and timescales.

The present results should be interpreted in light of several technical limitations. First, our experiments cannot fully capture the complex network interactions that likely contribute to *in vivo* BFCN responses. The BFCN-induced onset spiking (Fig. 4) observed in L6 RS units is consistent with nAChR expression in these neurons (Fig.  2), while the slower effects align with mAChR transcript expression (Fig.  3), as supported by our *in vitro* results (Fig.  5). However, *in vivo* dynamics may reflect indirect circuit mechanisms, such as L1-IN-mediated disinhibition (Letzkus et al. 2011; Pi et al. 2013). As more data emerge on cell type–specific cholinergic effects, future models that integrate both receptor- and circuit-level mechanisms will better explain these responses. Second, our *in vivo* approach measures only spiking activity and therefore cannot capture subthreshold changes in membrane potential. Important prior work shows that L5-PNs across cortical regions, including ACtx, express both nAChRs and mAChRs and exhibit ACh-induced depolarization and hyperpolarization (Gulledge and Stuart 2005; Hedrick and Waters 2015; Joshi et al. 2016). The absence of a significant effect time-locked to the laser onset in our L5 RS units may thus reflect limited sensitivity to subthreshold events. Finally, our recordings did not distinguish between the diverse L6-PN populations, which include both non-CT neurons and multiple CT subtypes distributed across L6 that project to distinct thalamic targets (Qi et al. 2025). Future studies will be needed to determine whether subtypes of L6-PNs exhibit differential cholinergic modulation in ACtx.

Together, our findings identify L6 of primary ACtx as a critical site for cholinergic modulation, where pyramidal neurons integrate fast and slow ACh signals via nAChRs and mAChRs. These interactions operate across distinct temporal and spatial scales, supporting a model in which ACh dynamically sculpts cortical computations through finely tuned layer- and cell type-specific mechanisms. This work underscores the importance of incorporating deep-layer circuits into frameworks of neuromodulation and sensory plasticity.

## Supplementary Material

Supplementary_materials_bhaf338
