# Supplementary material for: Layer 6 is a hub for cholinergic modulation in the mouse auditory cortex
Source: Cereb Cortex. 2026 Jan 22;36(1):bhaf338. doi: 10.1093/cercor/bhaf338 (PMC12825313; doi:10.1093/cercor/bhaf338)
Supplement: Supplementary_materials_bhaf338 [file supplementary_materials_bhaf338.docx]

**Supplementary Material**

Layer 6 is a hub for cholinergic modulation in the mouse auditory cortex

Lucas G. Vattino^1,2,4^, Kameron K. Clayton^1,2,4^, Troy A. Hackett^3,4^, Daniel B. Polley^1,2,*^, Anne E. Takesian^1,2,*^

*^1^Eaton-Peabody Laboratories, Massachusetts Eye and Ear, Boston, MA, 02114, USA ^2^Department of Otolaryngology - Head and Neck Surgery, Harvard Medical School, Boston, MA, 02115, USA*

*^3^Vanderbilt School of Medicine, Department of Hearing and Speech Sciences, Vanderbilt University Medical Center, Nashville, TN, 37232, USA*

*^4^These authors contributed equally*

*^*^Co-corresponding authors:* [*anne_takesian@meei.harvard.edu*](mailto:anne_takesian@meei.harvard.edu) [*daniel_polley@meei.harvard.edu*](mailto:daniel_polley@meei.harvard.edu)*; Massachusetts Eye and Ear, 243 Charles St, Boston, MA, 02114, USA*

**Table S1 – related to Figure 1D.** Total number of transcripts for nAChR subunits or mAChRs across layers in ACtx.

| **Total number of transcripts (N = 3 mice)** | **L1** | **L2/3** | **L4** | **L5** | **L6** |
| --- | --- | --- | --- | --- | --- |
| **α4** | 1715.67  ±  316.53 | 2684.67  ±  331.44 | 3910.67  ±  365.88 | 14232.33  ±  916.99 | 31700.33  ±  1501.88 |
| **α7** | 1456.33  ±  35.04 | 3880.67  ±  696.29 | 5769.67  ±  1362.55 | 6632.00  ±  1337.14 | 12012.00  ±  2770.313 |
| **β2** | 986.67  ±  179.74 | 9845.00  ±  962.41 | 14793.33  ±  2220.30 | 13397.33  ±  1980.20 | 15225.33  ±  1789.46 |
| **M1** | 181.00  ±  46.64 | 21613.67  ±  3688.20 | 18406.33  ±  3303.23 | 13663.33  ±  1201.17 | 22662.67  ±  2629.09 |
| **M2** | 21.67  ±  10.63 | 2267.00  ±  333.16 | 11061.00  ±  906.33 | 5206.67  ±  353.60 | 4875.00  ±  227.50 |
| **M3** | 398.67  ±  64.91 | 13644.33  ±  687.56 | 8208.00  ±  421.91 | 8078.33  ±  514.97 | 16139.00  ±  1185.99 |
| **M4** | 106.67  ±  38.90 | 6569.00  ±  840.07 | 12240.67  ±  2337.579 | 7147.67  ±  1180.10 | 10009.33  ±  1815.24 |

**Table S2 – related to Figure 2B and Figure 3B.** Fraction of VGluT1^+^ or VGAT^+^ cells expressing mAChRs or the different combinations of subunits for nAChRs.

| **Fraction of cells (N = 3 mice)** | | **L1** | **L2/3** | **L4** | **L5** | **L6** |
| --- | --- | --- | --- | --- | --- | --- |
| **VGluT1^+^** | **α4+β2** | - | 2.40 ± 0.36 | 2.99 ± 0.69 | 34.98 ± 1.05 | 26.41 ± 3.17 |
|  | **α7** | - | 0.66 ± 0.23 | 0.34 ± 0.14 | 1.10 ± 0.17 | 1.19 ± 0.10 |
|  | **α4+β2+α7** | - | 0.71 ± 0.58 | 1.91 ± 1.20 | 16.04 ± 4.21 | 29.03 ± 6.68 |
|  | **M1** | - | 87.38 ± 4.06 | 72.83 ± 5.78 | 72.35 ± 5.89 | 78.00 ± 4.74 |
|  | **M2** | - | 10.28 ± 2.48 | 53.79 ± 4.30 | 29.71 ± 3.97 | 13.13 ± 1.27 |
|  | **M3** | - | 69.33 ± 4.79 | 31.87 ± 0.65 | 44.42 ± 1.89 | 62.07 ± 1.64 |
|  | **M4** | - | 32.70 ± 5.51 | 50.46 ± 9.39 | 39.50 ± 5.84 | 36.17 ± 8.50 |
| **VGAT^+^** | **α4+β2** | 12.12 ± 9.90 | 17.10 ± 8.27 | 16.22 ± 1.98 | 17.92 ± 3.57 | 20.02 ± 5.16 |
|  | **α7** | - | - | - | - | - |
|  | **α4+β2+α7** | 75.17 ± 1.19 | 20.41 ± 5.99 | 8.73 ± 3.94 | 11.71 ± 1.54 | 8.91 ± 2.30 |
|  | **M1** | - | 26.20 ± 10.61 | 21.30 ± 9.37 | 14.55 ± 1.52 | 12.45 ± 4.48 |
|  | **M2** | - | 2.22 ± 1.81 | 6.68 ± 2.74 | 14.15 ± 2.03 | 30.28 ± 4.74 |
|  | **M3** | 7.41 ± 6.05 | 31.83 ± 5.56 | 29.58 ± 3.21 | 26.17 ± 3.04 | 37.61 ± 5.69 |
|  | **M4** | - | 14.85 ± 2.95 | 26.22 ± 9.11 | 17.89 ± 3.87 | 29.30 ± 4.53 |

**Table S3 – related to Figure 2C and Figure 3C.** Fraction of CTB^+^ and CTB^-^ cells expressing mAChRs or the different combinations of nAChR subunits.

| **Fraction of cells (N = 3 mice)** | **α4+β2** | **α7** | **α4+β2+α7** | **M1** | **M2** | **M3** | **M4** |
| --- | --- | --- | --- | --- | --- | --- | --- |
| **CTB^+^** | 29.16  ±  2.99 | - | 33.78  ±  5.98 | 84.75  ±  5.59 | 7.17  ±  1.69 | 68.67  ±  1.70 | 40.86  ±  8.83 |
| **CTB^-^** | 22.21  ±  2.81 | 2.82  ±  0.53 | 22.62  ±  6.38 | 70.64  ±  5.95 | 20.36  ±  3.70 | 55.43  ±  1.91 | 30.86  ±  8.48 |
| **CI 95% (lower,upper)** | CTB^+^: (13.37,44.94)  CTB^-^: (7.41,37.2) | CTB^+^: N/A  CTB^-^: (0.02, 5.63) | CTB^+^: (2.28,65.28)  CTB^-^: (1.02,56.26) | CTB^+^: (55.28,114.20)  CTB^-^: (39.27,102.00) | CTB^+^: (1.75,16.08)  CTB^-^: (0.88,39.84) | CTB^+^: (59.74,77.60)  CTB^-^: (45.37,65.48) | CTB^+^: (5.65,87.37) CTB^-^: (13.82,75.54) |
| **Effect sizes (Cohen’s d)** | 1.13 | N/A | 0.85 | 1.16 | 2.16 | 3.46 | 0.55 |

**Table S4 – related to Figure 4D, E.** Statistical comparison between Baseline, Onset and Persistent for RS units in the different layers.

| Mean absolute Z-score (N = 4 mice) | Baseline | Onset | Persistent | Mixed-effects one-way ANOVA nested within mice, with Tukey’s post-hoc comparisons |
| --- | --- | --- | --- | --- |
| L2/3 (n = 17 units) | 0.40 ± 0.06 | 0.57 ± 0.09 | 0.94 ± 0.07 | F2,9 = 5.65, p = 0.03; Baseline vs. Onset: p = 0.61; Baseline vs. Persistent: p = 0.02; Onset vs. Persistent: p = 0.11 |
| L4 (n = 18 units) | 0.39 ± 0.06 | 0.54 ± 0.12 | 0.96 ± 0.05 | F2,9 = 5.23, p = 0.03; Baseline vs. Onset: p = 0.71; Baseline vs. Persistent: p = 0.03; Onset vs. Persistent: p = 0.10 |
| L5 (n = 51 units) | 0.35 ± 0.04 | 0.47 ± 0.05 | 0.95 ± 0.04 | F2,9 = 14.63, p = 0.002; Baseline vs. Onset: p = 0.55; Baseline vs. Persistent: p = 0.002; Onset vs. Persistent: p = 0.007 |
| L6 (n = 59 units) | 0.29 ± 0.02 | 0.66 ± 0.07 | 0.96 ± 0.04 | F2,9 = 9.604, p = 0.0059; Baseline vs. Onset: p = 0.04; Baseline vs. Persistent: p = 0.005; Onset vs. Persistent: p = 0.33 |

**Table S5 – related to Figure 5.** Statistical comparison of the intrinsic properties for the neurons recorded during *in vitro* whole-cell experiments, showing differential cholinergic responses.

|  | L6-PNs depolarizing (n = 12 cells) | L6-PNs biphasic (n = 5 cells) | L6-PNs hyperpolarizing (n = 5 cells) | L6-PNs non-responding (n = 3 cells) | One-way ANOVA with Fisher’s post-hoc comparisons |
| --- | --- | --- | --- | --- | --- |
| **Passive properties** | | | | | |
| RMP (mV) | -65.66 ± 0.95 | -66.43 ± 0.87 | -66.43 ± 1.36 | -65.98 ± 1.64 | F_3,20_ = 0.12, p = 0.95 |
| R_input_ (MOhm) | 300.80 ± 37.46 | 327.70 ± 26.27 | 258.60 ± 53.81 | 309.90 ± 46.62 | F_3,20_ = 0.30, p = 0.83 |
| C_m_ (pF) | 17.31 ± 0.49 | 16.82 ± 0.94 | 16.58 ± 0.61 | 17.68 ± 1.37 | F_3,20_ = 0.1182, p = 0.95 |
| Tau (ms) | 34.79 ± 2.32 | 44.69 ± 4.46 | 38.76 ± 6.54 | 35.75 ± 3.64 | F_3,20_ = 1.42, p = 0.27 |
| **Repetitive firing (during 200 pA step)** | | | | | |
| Action potential threshold (mV) | -45.94 ± 1.49 | -47.92 ± 3.3 | -40.71 ± 5.19 | -49.00 ± 5.44 | F_3,20_ = 1.03, p = 0.40 |
| Action potential latency (ms) | 10.03 ± 1.36 | 11.32 ± 2.40 | 8.57 ± 2.06 | 12.47 ± 0.78 | F_3,20_ = 0.52, p = 0.68 |
| Action potential height (mV) | 52.98 ± 3.40 | 56.14 ± 3.64 | 51.91 ± 7.90 | 54.00 ± 5.39 | F_3,20_ = 0.12, p = 0.95 |
| Action potential half-width (ms) | 3.88 ± 0.78 | 4.53 ± 0.34 | 3.71 ± 0.26 | 3.63 ± 0.36 | F_3,20_ = 0.18, p = 0.91 |
